# Discrimination and hypertension among a diverse sample of racial and sexual minority men living with HIV: baseline findings of a longitudinal cohort study

**DOI:** 10.1038/s41371-024-00919-0

**Published:** 2024-06-26

**Authors:** Avrum Gillespie, Rui Song, John P. Barile, Lorie Okada, Shari Brown, Kerry Traub, Julia Trout, Gina M. Simoncini, Casey D. Xavier Hall, Yin Tan, Crystal A. Gadegbeku, Grace X. Ma, Frank Y. Wong

**Affiliations:** 1 Division of Nephrology, Hypertension, and Kidney Transplantation, Department of Medicine, Lewis Katz School of Medicine, Temple University, Philadelphia, PA USA; 2 Center for Asian Health, Lewis Katz School of Medicine, Temple University, Philadelphia, PA USA; 3 Department of Psychology, University of Hawaiʽi at Mānoa, Honolulu, HI USA; 4 Sidney Kimmel Cancer Center, Thomas Jefferson University, Philadelphia, PA USA; 5 Absolute Care, Philadelphia, PA USA; 6 Center of Population Sciences for Health Equity, College of Nursing, Florida State University, Tallahassee, FL USA; 7 Glickman Urological and Kidney Institute, Cleveland Clinic, Cleveland, OH USA

**Keywords:** Hypertension, Risk factors, Diagnosis, Medical research

## Abstract

Racial and sexual orientation discrimination may exacerbate the double epidemic of hypertension (HTN) and HIV that affects men of color who have sex with men (MSM). This was a cross-sectional analysis of African American, Asian American, Native Hawaiian, or Pacific Islander (NHPI) MSM living with HIV (PLWH) cohort in Honolulu and Philadelphia. Racial and sexual orientation discrimination, stress, anxiety, and depression were measured with computer-assisted self-interview questionnaires (CASI). We examined the associations between racial and sexual orientation discrimination with hypertension measured both in the office and by 24-h ambulatory blood pressure monitoring (ABPM) using multivariable logistic regression. Sixty participants (60% African American, 18% Asian, and 22% NHPI) completed CASIs and 24-h ABPM. African American participants (80%) reported a higher rate of daily racial discrimination than Asian American (36%) and NHPI participants (17%, *p* < 0.001). Many participants (51%) reported daily sexual orientation discrimination. Sixty-six percent of participants had HTN by office measurement and 59% had HTN by 24-h ABPM measurement. Participants who experienced racial discrimination had greater odds of having office-measured HTN than those who did not, even after adjustment (*Odds Ratio* 5.0 (95% Confidence Interval [1.2–20.8], *p* = 0.03)). This association was not seen with 24-h ABPM. Hypertension was not associated with sexual orientation discrimination. In this cohort, MSM of color PLWH experience significant amounts of discrimination and HTN. Those who experienced racial discrimination had higher in-office blood pressure. This difference was not observed in 24-h APBM and future research is necessary to examine the long-term cardiovascular effects.

## Introduction

A double epidemic of hypertension (HTN) and HIV affects men of color who have sex with men (MSM). People living with HIV (PLWH) have greater rates of HTN than those without the disease [1]. The biological mechanisms of HTN in HIV include endothelial dysfunction and the adverse effects of antiretroviral therapy [2]. Meanwhile, among PLWH of color in the United States (U.S.), the stress of racial discrimination may also increase blood pressure (BP) [3–5]. Furthermore, racial minority MSM are often exposed to homophobia [6], causing stress that could also increase BP.

Currently, there is no systematic study addressing how these factors influence HTN among African American, Asian American, and Native Hawaiian or Pacific Islander (NHPI) MSM living with HIV – three racial groups that are disproportionally affected by the HIV epidemic in the U.S. For example, MSM composed 37% of new HIV diagnoses in African Americans [7], 89% in Asian Americans [8], and 84% in NHPIs, respectively [9].

The 24-h ambulatory BP monitor (ABPM), often under-utilized in clinical practice, can more accurately predict target organ damage and cardiac risk compared to office BP [10, 11]. ABPM not only differentiates between sustained HTN, white-coat HTN, and masked HTN but also detects the normal physiologic nocturnal BP dip (10% drop in average BP during the nighttime compared to daytime) [11, 12]. The nocturnal dip is a phenomenon associated with circadian sympathetic rhythm [13] and normotensive or hypertensive individuals with a blunted “dipping” response are at increased risk for target organ damage and cardiovascular mortality [11].

African Americans and Asian Americans have been shown to have higher rates of non-dipping compared to whites [14, 15]. Several studies with African American participants have found that low or reverse nocturnal dip was associated with stress-inducing social factors including poverty [16], being unmarried, low education status [14], post-traumatic stress disorder [16], low perceived social support [14, 15], and everyday discrimination [17]. The research linking social factors to the high non-dipping rate in Asian Americans and NHPIs is limited. There is also a lack of evidence on the association between HTN and racial and sexual orientation discrimination among MSM of color living with HIV.

**Fig. 1:**
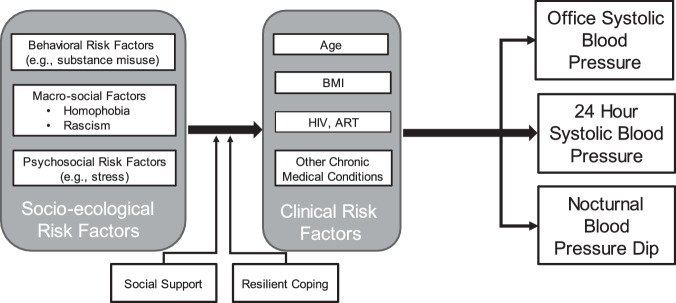
Conceptual framework of socio-ecological risk factors, clinical risk factors, health-related quality of life, and systolic blood pressure. The conceptual framework of the study in which we hypothesize that socio-ecological risk factors exacerbate clinical risk factors which can affect health-related quality of life and hypertension. We also hypothesize that social support and resilience can reduce the effects of these risk factors. Antiretroviral therapy (ART).

Given the higher rates of HTN and potential for psychosocial stress from discrimination among individuals, we report the baseline analysis of a longitudinal HIV and HTN cohort study examining the relationship of discrimination, psychosocial stress, and HTN on Asian American and NHPI MSM living with HIV in Hawai’i as well as African American MSM living with HIV in Philadelphia (see Fig. 1). Hawaiʽi was chosen because of its prevalent Asian (42.9%) and NHPI population (9.6%) who despite their plurality still experience systematic racism and discrimination [18, 19]. Philadelphia was chosen for its prevalent African American population (41.4%) who also despite their plurality, experience systematic racism and discrimination. No white MSM were part of this study because the goal of the study was to look at the intersection of race and sexual discrimination among racial minority groups [20]. We hypothesize that there is an additive effect of these stressors on hypertension and non-dipping.

## Methods

### Study design and setting

Data were derived from baseline assessments of an ongoing longitudinal HIV and HTN cohort study in Hawai‛i and Philadelphia. Eligible participants were recruited at clinics affiliated with our respective universities and governmental and non-governmental organizations such as AIDS Service Organization who serve MSM. Data were collected between May 2019 and August 2020. This study was performed in line with the principles of the Declaration of Helsinki and approved by the University of Hawai’i institutional review board.

### Participants

To be eligible for all activities, a participant must (1) have been age 18 or above (2) self-identify as African American, Asian, Native Hawaiian, or Other Pacific Islander, (3) self-identify as a biological male; (4) self-report having had sex with men in the last 12 months (oral, anal, or both); (5) a verifiable HIV-positive status; (6) have been able to give verbal and written consent; and (7) have been intending to stay in local study site area (Hawaiʽi or Philadelphia) for the next 36 months.

### Procedures

After consent was obtained, each participant: (1) completed a computer-assisted self-interviewing (CASI) based psychosocial and behavioral assessment and (2) learned how to use the ABPM device. Participants were compensated $20 for completing the CASI survey and use of the ABPM.

### Data sources: measurement

The primary outcome measures were HTN measured in the office, HTN by 24-h ABPM, and nocturnal dip. The primary exposures were sexual orientation discrimination and racial discrimination. Potential confounders included location and number of comorbidities.

#### Socio-demographic characteristics

Socio-demographic variables included age, race/ethnicity, family characteristics, occupation, family income, education, marital status, and neighborhood characteristics. Sex was measured by the question, “Do you self-identify as biological male?”

#### Psychosocial measures

The CASI-based assessment collected the following psychosocial measures: internalized homophobia scale [6], experience of discrimination scale [21], substance use/misuse, Center of Epidemiologic Studies Depression Scale [22], Perceived Stress Scale [23], Overall Depression Severity and Impairment Scale (OASIS) [24], and generalized anxiety disorder scale [25].

#### Health history

The CASI-based assessment included participants’ history of hypertension, their history family history of hypertension, and the Multiple Chronic Health Conditions Inventory [26]. The participants’ viral loads, heights, and weights were obtained from the electronic medical record.

##### ABPM assessment

Immediately after the completion of the CASI-based assessment, participants engaged in 24-h ABPM monitoring using the Welch-Allyn or SpaceLabs® system. After placing the monitor with the patient seated for 5 min per AHA standard guidelines [27], 3 readings were recorded at 3-min intervals. Subsequently, the ABPM monitor obtained readings at 30-min intervals. The ABPM was considered adequate and included for analysis if the monitor had been worn for a full 24 h and if there were at least 10 acceptable daytime readings and 5 acceptable nighttime readings according to the International Database of Ambulatory BP in relation to Cardiovascular Outcome (IDACO) criteria [28]. Data from the monitor were downloaded locally, deidentified, and transmitted electronically for analysis. The definition of daytime and nighttime is based on the self-reported activity log, which is thought to be more reliable than fixed time intervals [29]. For intact BP data without available activity logs, we used 9 AM–9 PM as daytime and 1 AM–6 AM as nighttime. The BP readings are further stratified into different phenotypes for comparison. Dipping status is calculated as 1 the ratio of the average systolic BP from nighttime divided by daytime. We defined individuals with nocturnal systolic BP dipping of less than 10% as “non-dipper.” [30] Hypertension was defined as having an office BP ≥ 130/80 mmHg, and/or average 24-h ABPM readings ≥125/75 mmHg [30]. Masked hypertension refers to a normal office BP with hypertension on ABPM reading and white-coat hypertension refers to hypertension in the office with normal ABPM readings.

### Analytic strategies

Categorical and dichotomous variables are presented as frequency counts and percentages; continuous variables are summarized by their mean and standard deviation (*SD*) or median and interquartile range (*IQR*), as appropriate. Two-tailed bivariate analyses were used as appropriate.

Five multivariable Firth logistic regression models were created with racial discrimination as the main variable of interest. Firth logistic regression was used based on the sample size [31]. Model 1 tests the univariate association with the dependent variable of office-based hypertension. Model 2 adds the study site and Model 3 adds the number of comorbidities. For Model 4, the dependent variable was 24-h SBP. For Model 5, the dependent variable was a nocturnal dip. We inspected the model residual plots for normality and homoscedasticity. SPSS version 27 [32] was used for the descriptive and bivariate and STATA 15 [33] was used for multivariable analyses.

Two sensitivity analyses were performed using bootstrap replication. The first sensitivity analysis was a bootstrap with 1000 replications performed on the association of daily racial discrimination and office-measured hypertension. The second analysis separated the data into each site (Hawaii and Philadelphia) and ran a bootstrap univariate logistic regression with 1000 replications for each site.

## Results

### Socio-demographic and clinical characteristics

Seventy-five participants were enrolled, 60 (80%) completed their questionnaire and ABPM in accordance with the IDACO criteria and were included in the analysis. Table 1 presents key socio-demographic characteristics of the sample. Thirty-six participants were recruited and enrolled in Philadelphia, and 24 were in Hawaiʽi with 23 from the island of Oʽahu and 1 from the island of Hawaiʽi.

**Table 1:** Sociodemographics and self-reported clinical data by race and ethnicity.

	Black N = 36 (59%)	Asian N = 11 (19%)	NHPI N = 13 (22%)	Total N = 60	*P* value
Age mean (SD)	48 (12)	48 (13)	52 (13)	49 (12)	0.67
Level of education					0.73
*Grade 12 or less*	10 (28%)	2 (18%)	3 (23%)	15 (25%)	
*Some college*	18 (50%)	4 (36%)	5 (39%)	27 (45%)	
*Bachelor’s degree or higher*	8 (22%)	5 (45%)	5 (39%)	18 (30%)	
Employment					**0.06**
*Full-time*	14 (39%)	4 (36%)	3 (23%)	21 (35%)	
*Part-time*	5 (14%)	1 (9%)	0 (0%)	6 (10%)	
*Self-employed*	2 (6%)	2 (18%)	0 (0%)	4 (7%)	
*Retired*	0 (0%)	2 (18%)	1 (8%)	3 (3%)	
*On disability*	11 (31%)	1 (9%)	4 (31%)	16 (27%)	
*Not employed*	4 (11%)	1 (9%)	5 (39%)	10 (17%)	
Income <40 K	32 (89%)	6 (55%)	7 (54%)	45 (75%)	**0.01**
Married or cohabiting	4 (11%)	5 (45%)	1 (8%)	10 (17%)	**0.02**
Substance use	20 (57%)	5 (45%)	6 (46%)	31 (53%)	0.69
Family history of HTN	19 (54%)	7 (64%)	5 (45%)	31 (54%)	0.69
Self – HTN	17 (47%)	2 (18%)	3 (25%)	22 (37%)	0.13
Taking HTN medicines	13 (36%)	2 (18%)	3 (25%)	18 (31%)	0.47
Heart disease	3 (9%)	1 (9%)	1 (9%)	5 (9%)	0.99
High cholesterol	12 (33%)	4 (36%)	3 (23%)	19 (32%)	0.74
Chronic pain	9 (25%)	1 (9%)	5 (39%)	15 (25%)	0.25
Diabetes	6 (17%)	2 (18%)	3 (23%)	11 (18%)	0.88
Depression	23 (64%)	6 (55%)	5 (39%)	34 (57%)	0.28
Anxiety	19 (53%)	3 (27%)	2 (15%)	24 (40%)	**0.04**
Sleep disorder	11 (31%)	3 (27%)	3 (25%)	17 (29%)	0.90
Hepatitis C	4 (12%)	0 (0%)	1 (8%)	4 (9%)	0.46
Height in. mean (SD)	71 (3)	67 (2)	68 (3)	69 (3)	**0.001**
Weight lbs. mean (SD)	182 (33)	151 (19)	179 (56)	175 (39)	**0.08**
BMI mean (SD)^a^	26 (4)	24 (2)	27 (7)	26 (5)	0.13
Suppressed viral load^b^	23 (96%)	4 (67%)	6 (100%)	33 (92%)	
CD4 mean (SD)^c^	631 (288)	863 (422)	472 (379)	628 (317)	0.25

*NHPI* Native Hawaiian or Pacific Islander, *SD* standard deviation, *40* *K* $40,000, *Hx* history, *HTN* hypertension, *BMI* body mass index.

*p* values < 0.10 are in bold.

^a^BMI was Missing in 5 participants.

^b^Viral load was missing in 22 participants, Viral load <200 is defined as suppressed.

^c^CD4 was missing in 28 participants.

The mean age of the participants was 49 ± 12 years old. Fifty-nine percent of the participants identified as Black or African American, 14% as Asian (Filipino, Chinese, Japanese, Korean, Okinawan), and 22% as NHPI. Six participants identified as multiethnic and were recorded based on their ethnic minority status. All the participants from Philadelphia identified as Black or African American. Three male participants who reported having sex with men identified as heterosexual.

Most participants (77%) reported at least one medical co-morbidity (Mean 5 ± 3, data not tabled). Thirty-seven percent reported having HTN and 32% reported high cholesterol. As for psychiatric comorbidities, 56% of participants reported having depression, 39% having anxiety, and 28% having a sleep disorder.

### BP measurements and HTN awareness by race

Table 2 presents BP measurements stratified by race. Asian participants had significantly lower in-office diastolic BPs (DBP), 24 ABPM, and nocturnal DBP resulting in lower rates of 24 ABPM hypertension when compared to African American and NHPI. African American participants had the highest in-office DBP whereas NHPIs had the highest 24 ABPM DBP. NHPIs also had the highest rates of hypertension (85%) as measured by 24 ABPM. Overall, 59% of participants had HTN measured by 24-h APBM and 54% were non-dippers.

**Table 2:** Association between self-reported race and ethnicity and blood pressure measurements.

Variables	Black N = 36 (60%)	Asian N = 11 (18%)	NHPI N = 13 (19%)	Total N = 60	*p value*
HTN measured					
In-office HTN	26 (72%)	4 (40%)	10 (77%)	40 (68%)	0.11
Office SBP mmHg	132 (13)	120 (14)	130 (17)	130 (14)	0.07
Office DBP mmHg	87 (11)	78 (10)	86 (9)	85 (11)	**0.05**
Aware of office HTN (n = 45)	17 (61%)	2 (33%)	3 (27%)	22 (49%)	0.12
24H ABPM HTN	21 (58%)	4 (36%)	11 (85%)	36 (60%)	**0.05**
24H ABPM SBP	120 (9)	117 (12)	127 (15)	121 (12)	0.10
24H ABPM DBP	75 (6)	74 (9)	82 (11)	77 (9)	**0.03**
Total wake hours	15 (3)	13 (3)	16 (4)	15 (3)	0.25
Wake SBP	125 (10)	122 (12)	129 (18)	126 (12)	0.38
Wake DBP	80 (7)	78 (9)	84 (13)	81 (9)	0.25
Sleep SBP	113 (12)	108 (13)	119 (16)	113 (13)	0.10
Sleep DBP	68 (8)	68 (9)	77 (12)	70 (10)	**0.02**
Aware of 24 ABPM HTN	17 (68%)	2 (33%)	3 (30%)	22 (54%)	0.07
White-coat HTN	8 (22%)	1 (10%)	2 (17%)	11 (19%)	0.67
Masked HTN	3 (8%)	1 (10%)	3 (25%)	7 (12%)	0.30
Non-dipper	19 (53%)	5 (45%)	8 (62%)	32 (53%)	0.73
% Nocturnal dip SBP	10 (7)	11 (7)	7 (10)	10 (8)	0.33
% Nocturnal dip DBP	14 (9)	12 (8)	8 (10)	10 (8)	0.10

24 h ambulatory blood pressure measurements, nocturnal dip in blood pressure. All values are Mean and Standard deviation unless otherwise specified.

*NHPI* Native Hawaiian or Pacific Islander, *SBP* systolic blood pressure, *DBP* diastolic blood pressure, *24H ABPM* 24-h ambulatory blood pressure monitoring.

*p* values < 0.10 are in bold.

Forty-five percent of participants with elevated in-office systolic BP (SBP) and 39% with elevated 24-h ABPM SBP were not aware of their hypertension and thus not taking anti-hypertensive medication (Table 2). Rates of hypertension awareness were the lowest among NHPI. Seventy percent of the NHPI participants with elevated in-office SBP were not receiving anti-hypertensive medication (data not in table). It should be noted that 8 participants, 12%, had masked HTN (Table 2).

### Discrimination and psychosocial measurements

As shown in Table 3, a greater percentage of Black participants reported daily racial discrimination compared to Asians and NHPIs and as a result, more participants in Philadelphia experienced discrimination than in Hawaii. The most common racial discrimination was being discriminated against on the street or in a public place (79%), followed around in a store (74%), and discriminated against at school (56%). Fifty-one percent of participants reported daily sexual discrimination. The most common sexual orientation discrimination was being discriminated against on the street or in a public place (73%), discriminated against at school (57%), and getting poor service at a store or restaurant (50%, data not in tables). Race was not associated with sexual discrimination. Participants who experienced racial and sexual discrimination also had more comorbidities (5.0 ± 3.7 vs. 2.5 ± 3.8, *p* = 0.002 and 5.0 ± 3.7 vs. 2.5 ± 2.6, *p* = 0.006; respectively). Participants who experienced racial discrimination scored higher on the Overall Anxiety Severity and Impairment Scale (OASIS) than those who did not (5.5 ± 4.8 vs. 3.0 ± 3.6, *p* = 0.02). This difference in the OASIS was not observed in participants who experienced sexual orientation discrimination. Racial and sexual orientation discrimination was not associated with a difference in the Center of Epidemiologic Studies (CES) depression scale, perceived stress, or Generalized Anxiety Disorder (GAD) scale.

**Table 3:** Racial and sexual orientation discrimination by demographics, psychosocial stress, and hypertension.

	Experienced daily racial discrimination^a^			Experienced daily sexual orientation discrimination		
	Yes N = 35 (59%)	No N = 24 (41%)	*p* value	Yes N = 31 (52%)	No N = 29 (48%)	*p* value
Site			<0.001			0.07
Philadelphia	29 (83)	7 (29)		22 (71)	14 (48)	
Hawaii	6 (17)	17 (71)		9 (29)	15 (52)	
Race/Ethnicity			<0.001			0.15
Black	29 (83)	7 (29)		22 (71)	14 (48)	
Asian	4 (11)	7 (29)		5 (16)	6 (21)	
NHPI	2 (6)	10 (42)		4 (13)	9 (31)	
Number comorbidities mean (SD)	5.0 (3.7)	2.5 (3.8)	0.002	5.0 (3.9)	2.7 (2.6)	0.006
Psychological measurements						
CES-depression scale mean (SD)	9.7 (6.5)	7.6 (6.8)	0.22	9.0 (6.5)	8.7 (6.9)	0.90
Perceived stress scale mean (SD)	15.4 (8.0)	13.6 (7.8)	0.38	15.3 (7.2)	14.0 (8.6)	0.53
OASIS mean (SD)	5.5 (4.8)	3.0 (3.6)	0.02	4.4 (4.1)	4.5 (5.0)	0.99
Generalized Anxiety Disorder Scale mean (SD)	5.3 (4.5)	3.8 (4.1)	0.18	5.0 (4.5)	4.3 (4.3)	0.56
Hypertension measurements^b^						
Office SBP mean (SD)	133 (12)	124 (16)	0.02	130 (11)	129 (16)	0.79
Office DBP mean (SD)	88 (10)	81 (11)	0.005	87 (11)	84 (12)	0.36
24-h ABPM SBP mean (SD)	120 (10)	121 (12)	0.80	122 (11)	120 (12)	0.69
24-h ABPM DBP mean (SD)	76 (8)	76 (8)	0.96	77 (7)	76 (10)	0.81
Nocturnal dip SBP (%) mean (SD)	11 (7)	9 (9)	0.35	9 (8)	11 (8)	0.23
Nocturnal dip DBP (%) mean (SD)	14 (9)	10 (9)	0.11	13 (10)	12 (9)	0.90
Office HTN	28 (80)	11 (48)	0.01	22 (71)	17 (63)	0.52
24 ABPM HTN	21 (60)	14 (58)	0.90	21 (68)	15 (52)	0.21
Non-dipper	18 (51)	13 (54)	0.59	19 (61)	13 (45)	0.20
White-coat HTN	8 (23)	3 (13)	0.50	5 (16)	6 (22)	0.74
Masked HTN	1 (3)	6 (27)	0.01	4 (13)	3 (11)	1.00
HTN awareness (n = 45,46)^c^	18 (62)	5 (31)	0.05	14 (56)	9 (43)	0.37

The demographic, psychological, and blood pressure differences in participants who experienced daily racial and daily sexual orientation discrimination. All categorical variables are represented as counts and (percentages). Continuous variables are represented as mean and (standard deviation).

*SD* standard deviation, *NHPI* Native Hawaiian or Pacific Islander, *SBP* systolic blood pressure, *DBP* diastolic blood pressure, *ABPM* ambulatory blood pressure monitoring, *OASIS* Overall Anxiety Severity and Impairment Scale, *CES* Center of Epidemiologic Studies.

^a^One participant did not answer the question about daily racial discrimination.

^b^One participant did not have his blood pressure measured in the office.

^c^This among participants who had an SBP > 130 in the office or a self-reported history of HTN.

### Discrimination and HTN

Participants who experienced daily racial discrimination had higher office systolic and diastolic BP compared to those who did not experience daily racial discrimination (SBP 133 ± 12 vs. 124 ± 16, *p* = 0.02 and DBP 88 ± 10 vs. 81 ± 11, *p* = 0.005; respectively). Thus, more participants who had experienced racial discrimination met the criteria for HTN by in-office BP measurement. This association between racial discrimination and HTN was not seen on 24-h ABPM SBP&DBP, and percent dip in nocturnal SBP. Daily sexual orientation was not associated with office-based and 24-h ABPM SBP nor nocturnal SBP dip.

Next, we created Firth logistic regression models to examine the contribution of other variables to office hypertension including the survey site and the number of comorbidities. Model 1 is the unadjusted model indicating that participants who experience daily racial discrimination had 4.4 greater odds (odds ratio *OR* 4.4 (95% confidence interval *CI* [1.4–14.0], *p* = 0.01, Table 4, Model 1)) of having hypertension than those who did not. Model 2 adjusts for the survey site and shows that office-measured HTN remained significantly associated with racial discrimination (*OR* 5.1 95%CI [1.2–20.1], *p* = 0.02; Model 2, Table 4) despite there being significantly more discrimination at the Philadelphia site than the Hawaii site. Next, we added the number of comorbidities, as this was significantly associated with discrimination and could explain hypertension. The number of comorbidities; however, was not associated with hypertension, and discrimination remained significantly associated with hypertension, but the model was no longer significant (Table 4, Model 3). We also adjusted the model for age and BMI and racial discrimination remained significantly associated with hypertension (see Supplemental Table 3). Neither hypertension as measured by 24 h ABPM (Table 4, Model 4) nor nocturnal dip (Table 4, Model 5) were associated with discrimination after multivariable adjustment.

**Table 4:** Associations of racial discrimination, site, and number of comorbidities with office systolic blood pressure, 24 h ABPM systolic blood pressure, and percent dip in systolic blood pressure.

Variables	Model 1 Office HTN Discrimination Univariate N = 58 *p* = 0.01, pll −31.7	Model 2 Office HTN Discrimination and Site N = 58, *p* = 0.05, pll −31.3	Model 3 Office HTN Discrimination, Site, and Comorbidities N = 58, *p* = 0.11, pll −28.7	Model 4 24H ABPM HTN Discrimination, Site, and Comorbidities N = 59, *p* = 0.96, pll −34.8	Model 5 Nocturnal Dip Discrimination, Site, and Comorbidities N = 59, *p* = 0.99, pll −35.8
Daily Racial Discrimination	4.1 [1.3, 12.9], 0.01	4.5 [1.2–17.2], 0.03	5.0 [1.2–20.8], 0.03	1.1 [0.3–3.8], 0.91	0.8 [0.2–2.8], 0.77
Hawaii (Site)		1.2 [0.3–4.8], 0.75	1,2 [0.3–4.7], 0.76	0.8 [0.2–2.8], 0.76	1.1 [0.3–3.7], 0.86
Number of Comorbidities			0.95 [0.8–1.1], 0.56	1.0 [0.9–1.2], 0.66	1.0 [0.9–1.2], 0.88

The associations presented as odds ratios with 95% confidence intervals between racial discrimination, site, and the number of comorbidities with in-office systolic hypertension (Model 1–3), Hypertension as measured by 24 h ambulatory blood pressure monitoring (Model 4), and whether there was less than a 10% dip in the nocturnal dip in systolic blood pressure (Model 5). A positive coefficient Model 5 represents the odds of systolic non-dipping.

*SBP* systolic blood pressure, *NHPI* Native Hawaiian or Pacific Islander, *ABPM* ambulatory blood pressure monitor, *HTN* hypertension, *pll* penalized log likelihood.

### Sensitivity analyses

Racial discrimination remained associated with office-measured hypertension after one thousand bootstrap replications (4.4 [1.3, 15.2], 0.02, Supplemental Table 2, Analysis 1). In analysis two, there was a similar positive association of racial discrimination and HTN when examined at Site 1, Hawaiʽi (5.0 [1.0–25.2], 0.05) and Site 2, Philadelphia (5.1 [0.8–32.2], 0.08); however, the associations were no longer significant after one thousand bootstrap replications.

## Discussion

We set out to examine the baseline burden of racial discrimination, sexual orientation discrimination, and HTN among MSM of color living with HIV. We found that most participants experienced racial discrimination (59%) and sexual orientation discrimination (51%). African American participants experienced the most racial discrimination. Participants also had a high burden of HTN (including an absence of a nocturnal dip). Racial discrimination was associated with higher BP in the office but not on 24-h APBM or nocturnal dip. There were no associations between sexual orientation discrimination and HTN.

We were surprised that only the office and not 24-h BP measurements were elevated in the context of exposure to racial discrimination. Previous studies have found a differential effect of racial discrimination on HTN, with lifetime discrimination increasing the incidence of HTN whereas daily reported discrimination did not [5, 6], suggesting that the long-term systemic effects of racism may be more impactful than the daily stressors. One explanation for the differences in office and 24H ABPM measurement could be office measurement error; however, if so, the effects would differ by site. Another explanation for differences in office and 24 ABPM is white-coat HTN. Thirty percent of those who had HTN in the office and experienced daily discrimination had white-coat HTN. White-coat hypertension has been associated with anxiety [34] and the racial discrimination participants experienced while coming to their clinical triggered their anxiety resulting in higher office BP measurements but the rest of the time, they avoided these daily triggers of racial discrimination and had normal BP measurements. Of note, white-coat HTN although initially thought to be benign, has been associated with increased cardiovascular-related deaths [12]. Thus, we will continue to follow this cohort for the long-term effects of racial discrimination on hypertension and cardiovascular outcomes.

Racial minorities are at an increased risk for both HIV and HTN resulting in a potential syndemic [35] and we examine the results of this cohort to previous studies. In our study, the prevalence of HTN among the study’s Asian cohort, 36%, was like the prevalence from a meta-analysis of 49 studies from the Americas, Europe, Africa, and Asia which estimated that 35% of HIV-positive patients receiving antiretroviral therapy have HTN [36]. We can also compare the prevalence of HTN in this study to studies of HIV-uninfected individuals. For example, 57% of African American participants with LWH had hypertension, which is similar to the Cardia study [5] which found that 50% of HIV uninfected African American participants have HTN by age 46 and 75% by age 55. In our study, NHPI participants living with HIV had the highest prevalence of HTN (85%). This is much higher than previous estimates of NHPI uninfected by HIV which were between 23% and 52% [37, 38]. Further research is needed to confirm this high prevalence.

In our study, the prevalence of non-dippers or reverse dippers was 68%. Similarly, a South African study of nocturnal dipping and HIV found the prevalence of non-dipping at 65% [39]. It should be noted that study’s sample was 91% female. A meta-analysis by Kent et al. [40]. of 8 studies also found less nocturnal dipping in HIV-positive individuals than in HIV-negative individuals. Previous studies have also linked systemic racism and the resulting post-traumatic stress disorder to non-dipping [16]; however, in our study, discrimination was not associated with loss of nocturnal dip.

These results must be interpreted in the context of their limitations. First, the study was limited by the sample size because recruitment was terminated early because of the COVID-19 pandemic. This minimized the number of variables we could simultaneously analyze in our conceptual model. While these are two very different geographical sites, levels of racial discrimination and discrimination may vary in other locations. Our sample size was also limited by excluding 21% of participants because of incorrect usage of ABPM. Because of these sample size limitations, our analysis combined those who were aware and treated for HTN and those who were unaware of their HTN diagnosis. Notably, those who experienced daily racial discrimination tended to be aware of their HTN and receiving treatment for HTN suggesting that the daily stressors of racial discrimination cause elevation in SBP despite treatment for HTN. It is unclear how much elevated systolic blood pressure is a direct physiologic response to discrimination and how much discrimination affects medication and lifestyle adherence [41]. Nonetheless, efforts should be made to increase hypertension awareness because only 45% of participants with an SBP greater than 130 mmHg [27], and 33% with an SBP > 140 mmHg were aware of their diagnosis of hypertension [42]. Interventions should examine both patient-level and provider-level barriers to hypertension awareness. Lastly, this study is a cross-sectional baseline analysis and further research is underway to examine how discrimination and inflammation affect long-term cardiovascular outcomes.

In conclusion, this study provides insights and baseline information about the complex interplay of psychosocial factors and cardiovascular disease among MSM of color living with HIV. Further, many participants with elevated BP were either unaware or undiagnosed with HTN. This finding identifies opportunities for health interventions to improve HTN awareness and treatment in these high cardiovascular-risk populations.

## Summary

### What is known about the topic

- A double epidemic of hypertension and HIV affects men of color who have sex with men.
- Men of color who have sex with men living with HIV in the United States experience significant racism and homophobia causing stress that could also increase blood pressure.
- The 24-h ambulatory BP monitor more accurately predicts target organ damage and cardiac risk compared to office blood pressure. It also detects the normal physiologic nocturnal BP dip which is more likely to be absent in those experiencing significant stress.

### What this study adds

- This cohort of African Americans, Asian Americans, Native Hawaiian, and Pacific Islander men who have sex with men living with HIV in Honolulu or Philadelphia experienced significant amounts of discrimination and hypertension.
- Those who experienced racial discrimination had higher in-office blood pressure. This difference was not observed with 24-h ambulatory blood pressure monitoring.
- Many participants with elevated blood pressure were either unaware or undiagnosed with hypertension. This finding identifies opportunities for health interventions to improve hypertension awareness and treatment in these high cardiovascular-risk populations.

### Supplementary information

Supplemental Material

## Data Availability

Deidentified data are available by request from Dr. Barile and approval of Principal investigator’s Drs. Ma and Wong as is permitted within the scope of the IRB protocol given the sensitive and protected nature of the data.

## Materials availability

Materials are available by request from Dr. Barile and approval of Principal investigator’s Drs. Ma and Wong as is permitted within the scope of the IRB protocol given the sensitive and protected nature of the data.

## Code availability

Code is available upon request.
