# Supplementary material for: Discrimination and hypertension among a diverse sample of racial and sexual minority men living with HIV: baseline findings of a longitudinal cohort study
Source: J Hum Hypertens. 2024 Jun 26;38(8):603–10. doi: 10.1038/s41371-024-00919-0 (PMC11329369; doi:10.1038/s41371-024-00919-0)
Supplement: Supplementary file 1 — Supplemental Material [file 41371_2024_919_MOESM1_ESM.docx]

**Supplemental Material**

**Table of Contents**

**Supplemental Table 1. Experienced Discrimination and Quality of Life..……………….……2**

**Supplemental Table 2. Bootstrap models of univariate logistic regression models examining the association of daily racial discrimination and office hypertension at both sites and each site individually……………………………………………………..………….…….3**

**Supplemental Table 3. Examining the association of daily racial discrimination, body mass index (BMI), age, and office hypertension……………………………………………..……4**

**Supplemental Table 4: Associations of Sexual Orientation Discrimination, Site, and Number of Comorbidities with Office Systolic Hypertension, 24 Hour ABPM Systolic Blood Pressure, and Percent Dip in Systolic Blood Pressure……………………………….…5**

**Supplemental Table 1. Experienced Discrimination and Quality of Life**

|  | Experienced Racial Discrimination | | | Experienced Sexual Orientation Discrimination | | |
| --- | --- | --- | --- | --- | --- | --- |
|  | Yes  N=29 | No  N=21 | *p* value | Yes  N=26 | No  N=24 | *p* value |
| In general my health is _____.^a^ | 3 (2-3) | 3 (2-3.5) | 0.52 | 3 (2 - 3) | 3 (2-3.7) | 0.49 |
| Number of days your physical health was not good (past 30 days) | 2 (0-9.5) | 1 (0-6) | 0.85 | 3 (0.7 – 9.3) | 0.5 (0 – 6.3) | 0.14 |
| Number of days your mental health was not good (past 30 days) | 7 (1.5-15) | 2 (0-5.5) | 0.5 | 4 (1 – 11.3) | 2.5 (0 – 12.5) | 0.14 |
| Number of days your physical or mental health kept you from doing your usual activities (past 30 days) | 5 (0.5 – 9.0) | 0 (0 - 0) | 0.02 | 1.5 (0 – 6.8) | 1 (0 – 8.5) | 0.20 |
| Number of days pain made it hard for you to do your usual activities (past 30 days) | 1 (0 - 9) | 1 (0 – 4.5) | 0.61 | 1.5 (0 – 8.3) | 1 (0 - 5) | 0.74 |
| Number of days you felt sad, blue, or depressed (past 30 days) | 7 (1 - 13) | 1 (0 - 6) | 0.03 | 6 (1 – 10.3) | 2 (0 - 13) | 0.14 |
| Number of days you felt worried, tense, or anxious (past 30 days) | 10 (1 - 15) | 3 (1 – 9.5) | 0.23 | 4 (1 - 15) | 4 (0.3 – 14.8) | 0.61 |
| Number of days you felt you did not get enough rest or sleep (past 30 days) | 8 (4 - 13) | 3 (0 – 6.5) | 0.02 | 6.5 (3.8 -10.5) | 5 (0.3 – 13.0) | 0.25 |
| Number of days you felt very healthy and full of energy (past 30 days) | 16 (6.5 – 26.5) | 22 (5 - 26) | 0.96 | 19.5 (6.8 – 27) | 18 (5 – 25) | 0.98 |

1. Self-reported health is a 5-point Likert scale with 1. Excellent, 2. Very Good. 3. Good, 4. Fair, 5. Poor.

**Supplemental Table 2. Bootstrap models of univariate logistic regression models examining the association of daily racial discrimination and office hypertension at both sites and each site individually.**

| Variables | Model 1 Both Sites Bootstrap 1000 replications  Office SBP  n = 58  Pseudo R^2^ 0.09, *p* = 0.02 | Model 2, Hawaii Site Bootstrap 1239 replications  n = 22  Pseudo R^2^ 0.07, *p* = 0.05 | Model 3, Philadelphia Site  Bootstrap 1,392 Replications  n=36  Pseudo R^2^ 0.08, *p* = 0.08 |
| --- | --- | --- | --- |
| Daily Discrimination  Racial | 4.4 [1.3, 15.2], 0.02 | 5.0 [1.0 – 25.2], 0.05 | 5.1 [0.8 – 32.2], 0.08 |
| Constant | 0.9 [0.4-2.2], 0.84 | 1.0 [0.4-2.9], 1.00 | 0.7 [0.2-3.5], 0.71 |

Supplemental table 2 shows the coefficients are odds ratio and 95% confidence intervals for daily racial discrimination and office hypertension after 1000 bootstrap replications.

**Supplemental Table 3. Examining the association of daily racial discrimination, body mass index (BMI), age, and office hypertension.**

| Variables | n = 55  Pseudo R^2^ 0.20, *p* = 0.04 |
| --- | --- |
| Daily Discrimination  Racial | 4.2 [1.2, 14.7], 0.03 |
| BMI | 1.1 [0.97-1.3], 0.12 |
| Age | 0.8 [0.96-1.1], 0.80 |
| Constant | 0.04 [0.0-2.7], 0.13 |

Supplemental Table 3. shows the associations presented as odds ratios with 95% confidence intervals between discrimination, BMI, age with in office systolic blood pressure.

**Table 4: Associations of Sexual Orientation Discrimination, Site, and Number of Comorbidities with Office Systolic Hypertension, 24 Hour ABPM Systolic Blood Pressure, and Percent Dip in Systolic Blood Pressure**

| **Variables** | | | **Model 1 Office HTN Discrimination Univariate**  N=59 *p* = 0.59, pll -35.1 | **Model 2 Office HTN**  **Discrimination and Site**  N=59, *p* = 0.63, pll -34.2 | **Model 3 Office HTN**  **Discrimination, Site, and Comorbidities**  N = 59, *p* = 0.82, pll -31.8 | **Model 4 24H-ABPM HTN**  **Discrimination, Site, and Comorbidities**  N = 60, *p* = 0.61, pll -34.4 | **Model 5 Nocturnal Dip**  **Discrimination, Site, and Comorbidities**  N = 60, *p* = 0.60, pll =-35.4 |
| --- | --- | --- | --- | --- | --- | --- | --- |
| Daily Sexual Orientation  Discrimination | | 1.3 [0.5, 3.9], 0.59 | | 1.2 [0.41 – 3.6], 0.72 | 1.2 [0.4 – 3.8], 0.73 | 2.0 [0.7 -6.1], 0.21 | 2.2 [0.7 -6.3], 0.17 |
| Hawaii (Site) | |  | | 0.6 [0.2 – 1.9], 0.42 | 0.6 [0.2 – 1.9], 0.43 | 0.7 [0.2-2.1], 0.55 | 0.8 [0.3-2.4], 0.75 |
| Number of Comorbidities |  | | |  | 1.00 [0.7 – 6.6], 0.97 | 1.0 [0.9-1.2], 1.0 | 1.0 [0.8-1.1], 0.64 |

Table 4. shows the associations presented as odds ratios with 95% confidence intervals between sexual orientation discrimination, site, and number of comorbidities with in office systolic hypertension (Model 1-3), Hypertension as measured by 24 hour ambulatory blood pressure monitoring (Model 4) and whether there was less than a 10 percent dip in nocturnal dip in systolic blood pressure (Model 5). A positive coefficient Model 5 represents the odds of systolic non-dipping. Systolic blood pressure (SBP). Ambulatory blood pressure monitor (ABPM). Hypertension (HTN). Penalized log likelihood (pll).
